# Role of the mtDNA Mutations and Mitophagy in Inflammaging

**DOI:** 10.3390/ijms23031323

**Published:** 2022-01-25

**Authors:** Siarhei A. Dabravolski, Nikita G. Nikiforov, Alexander D. Zhuravlev, Nikolay A. Orekhov, Andrey V. Grechko, Alexander N. Orekhov

**Affiliations:** 1 Department of Clinical Diagnostics, Vitebsk State Academy of Veterinary Medicine [UO VGAVM], 7/11 Dovatora Str., 210026 Vitebsk, Belarus; 2 AP Avtsyn Research Institute of Human Morphology, 3 Tsyurupa Street, 117418 Moscow, Russia; nikiforov.mipt@googlemail.com (N.G.N.); zhuravel17@yandex.ru (A.D.Z.); 3 Center for Precision Genome Editing and Genetic Technologies for Biomedicine, Institute of Gene Biology, Russian Academy of Sciences, 34/5 Vavilova Street, 119334 Moscow, Russia; 4 Laboratory of Angiopathology, Institute of General Pathology and Pathophysiology, Russian Academy of Medical Sciences, 125315 Moscow, Russia; 5 Institute for Atherosclerosis Research, Osennyaya Street 4-1-207, 121609 Moscow, Russia; fuper@gmail.com (N.A.O.); a.h.opexob@gmail.com (A.N.O.); 6 Federal Research and Clinical Center of Intensive Care Medicine and Rehabilitology, 14-3 Solyanka Street, 109240 Moscow, Russia; avg-2007@yandex.ru

**Keywords:** mitophagy, ageing, inflammation, inflammaging

## Abstract

Ageing is an unavoidable multi-factorial process, characterised by a gradual decrease in physiological functionality and increasing vulnerability of the organism to environmental factors and pathogens, ending, eventually, in death. One of the most elaborated ageing theories implies a direct connection between ROS-mediated mtDNA damage and mutations. In this review, we focus on the role of mitochondrial metabolism, mitochondria generated ROS, mitochondrial dynamics and mitophagy in normal ageing and pathological conditions, such as inflammation. Also, a chronic form of inflammation, which could change the long-term status of the immune system in an age-dependent way, is discussed. Finally, the role of inflammaging in the most common neurodegenerative diseases, such as Alzheimer’s and Parkinson’s, is also discussed.

## 1. Ageing Theories

Ageing is a continuous multi-factorial process characterised by a gradual decrease in physiological functionality, resulting in gradually increasing vulnerability of the organism to environmental factors and, eventually, in death. Usually, ageing correlates with a higher risk to different pathogens and diseases (mental, neurodegenerative, heart) cancer. Many theories have been suggested to explain the ageing process [1,2], most of it, in a broad overview, could be sorted into three main groups: programmed, damage, and vicious cycles. Programmed theory suggests a genetically-based limitation of the lifespan that depends on the regulation of different systems (DNA methylation [3,4], autophagy [5,6], telomeres [7,8], autoimmunity [9,10] and others); damage theory suggests that internal and external (environmental) factors affect the organism at various levels, and that results in the shortening of the lifespan (DNA damage [11], free-radicals [12], transposable elements [13] and others); vicious cycles theory suggests the activity of the positive feedback loops between different age-related diseases (in particular, atherosclerosis, hypertension, diabetes, Alzheimer’s (AD) and Parkinson’s), thus supporting and promoting each other [14,15]. The main difference of the inflammaging concept is a chronic progressive increase in the proinflammatory status. This phenomenon combines a decline in adaptive immunity (immunosenescence) and a rise in proinflammation [16]. This review is dedicated to the role of mitochondrial mutation and mitophagy in inflammaging. For the other aspects of the inflammaging theories, we wish to redirect readers to the cited papers.

### 1.1. Mitochondria Functions, Quality Control and Turnover

The primary function of mitochondria is to supply cells with ATP, produced by oxidative phosphorylation of various substrates [17]. Additionally, mitochondria store a high concentration of calcium, thus participating in calcium homeostasis: interplay with endoplasmic reticulum Ca^2+^ storage [18], activation of the secondary messengers’ signalling systems [19], cell cycle [20], proliferation [21], regulation of the respiratory bioenergetics [22]. Mitochondria also participate in steroid biosynthesis [23], hormonal signalling [24], apoptosis programmed cell death [25], immune signalling [26] and other processes.

During normal functioning, mitochondria are exposed to molecular damage due to protein oxidation; with time, damaged proteins are accumulated and cause loss of membrane potential. Mitochondrial quality control (MQC) pathways are several important cellular mechanisms keeping mitochondria functional:

- Mitochondrial biogenesis is a process of mitochondrial self-replication. Mitochondrial biogenesis could be activated by numerous developmental signals, different types of cellular stress or in response to environmental stimuli. PGC-1α (Peroxisome proliferator-activated receptor gamma coactivator 1-alpha) is the central regulator of mitochondrial biogenesis [27].
- Fission and fusion, the most basic mechanism, seems to be conserved from yeast to mammals [28]. Mitochondrial morphology is dynamic and regulated by complex protein machinery-fission and fusion. The fusion of the outer membrane is regulated by mitofusin 1 and 2, and the inner membrane-by the dynamin-like GTPase (OPA1) [29].
- Mitophagy is a specialised form of autophagy, selectively degrading damaged and malfunctioning mitochondria. Mitophagy plays an essential role in many cellular processes, such as mitochondrial turnover, cell differentiation and embryonic development, apoptosis and inflammation, adjusting mitochondrial numbers to actual cellular metabolic needs. Impaired mitophagy is associated with many neurodegenerative diseases, cancer, ageing and other pathological conditions [30].
- The next pathway of the MQC acts when mtDNA damage has occurred and includes several repairing mechanisms. Base excision repair (BER) is the main mitochondrial DNA repair pathway; it repairs DNA damage during normal functioning (caused by alkylation, deamination or oxidation). In the first step, substrate-specific DNA glycosylases recognise and remove modified bases. The second step generates an abasic site, further processed by an apurinic/apyrimidinic endonuclease (APE1). In the next step, DNA polymerase γ incorporates a single nucleotide into the gap (short-patch BER) or two-seven nucleotides (long-patch BER). On the final step, 5′ flap is cleaved by FEN-1 protein and ligated with DNA ligase III. Mitochondrial BER significantly contributes to longevity determination in mammals [31].
- Damaged mitochondrial proteins could be repaired and refolded back to their native state. Several heat shock proteins (HSP) located in the mitochondrial matrix (for example, Hsp22, Hsp60 and Hsp70) have been connected with extended lifespan and increased stress resistance [32,33].
- Mitochondrial unfolded protein response (UPRmt) activates upon an accumulation of the critical amount of misfolded or damaged proteins. UPRmt requires active bidirectional communication and proteins exchange between nucleus and mitochondria. ATFS-1/ATF5 is the best characterised UPRmt pathway. Normal transport of the leucine zipper protein ATFS-1 is interrupted by mitochondria stress and redirected to the nucleus, where it could interact with DVE-1 and UBL-5. UPRmt acts through two main mechanisms: chromatin remodeling and stimulating the expression of several mitochondrial chaperones (hsp-60, hsp-6 and protease clpp-1) [34].
- Too damaged proteins that could not be efficiently repaired or refolded, irreversible degraded from the cell by proteolysis [35]. There are several mitochondrial protease systems with specific localisation of its activity: cytosolic (ubiquitin/26S proteasome system (UPS)), inner membrane internal protease (AAA-protease complex) [36] and matrix protease (Lon) [37].

### 1.2. Mitochondrial Genome and Heteroplasmy

To understand the role of mtDNA’s mutations in inflammaging, it is necessary to describe the basic features of the mitochondrial genome. MtDNA is mostly maternally-inherited, with the size of the double-stranded circular genome about ~16.4 kb. Most mitochondrial proteins are encoded in the nucleus, folded and transported into the mitochondria by the specialised importing pathway. MtDNA encodes only a limited number of molecules: 11 mRNAs, 22 tRNA, two rRNAs, and translates 13 subunits of the OXPHOS system (oxidative phosphorylation) [38]. Because mtDNA lacks histones, nuclear-encoded nucleoprotein complexes protects mtDNA from the generated oxidants damage, where mitochondrial transcription factor A plays the leading role [39].

Different cells, tissues and organs have genetically diverse populations of mitochondria. Mitochondria are dynamic, continuously fusing and fissioning. Hundreds of mtDNA could be proliferated independently and, even, as a part of the mitochondrial network, move to the neighbouring cells. The number of mtDNA copies per individual mitochondrion varies and could reach 10 nucleoids [40]. This polypoid nature of the mtDNA allows unique mutations to arise or be inherited by an independent cellular lineage. Heteroplasmy is the presence of both mtDNA genomes (wild-type and mutant) within one cell. Recent research suggests that even different cells of one organ could have a distinct set of nucleotide mutations [41]. During cytokinesis, mitochondria are unequally distributed between daughter cells. The precise molecular mechanism of the mitotic segregation of mtDNA mutations is unknown, while cells, to inherit healthier organelles, could clearly distinguish and avoid aged or damaged organelles [42,43].

The intercellular mitochondrial transfer has been shown on many non-lineage-related cells and tissues [44]. It is presumed that the main purpose of this process is to replace malfunctional mitochondria in recipient cells [45], but with a broad cross-cell-type compatibility of mitochondrial transfer, the same pathway could also be used to spread mtDNA mutations into new cells and tissues [46]. Interestingly, some mutant mtDNA genotypes could expand faster, especially when they have some advantage over wild-type mtDNA. For example, mtDNA genotypes with a large deletion would have a shorter genome and replicate more quickly [47]. The highest level of positive selection of the mutant mtDNA could be observed in intensely proliferating and spreading cancer cells [48,49]. Additionally, different mtDNA polymorphism variants could have an advantage in particular cell types, nuclear, or environmental contexts [50].

## 2. Key Roles of Mitochondria in the Current Ageing Theory

### 2.1. ROS-Mediated Accumulation of mtDNA Mutations

One of the most elaborated ageing theories implies a direct connection between ROS-mediated mtDNA damage and mutations [51]. Normal ageing is accompanied by a gradual decline in activity of mitochondrial enzymes, phosphocreatine recovery time and average respiratory capacity per mitochondria, while ROS production is increased [52]. In turn, increased ROS production makes more severe damage to mtDNA with a higher number of mutations, and thus, closing a so-called vicious cycle because such damaged mitochondria release more ROS.

A low-level of mutant mitochondrial genomes is usually inherited by a child from a mother and seems normal, while the number of mutations grows with age and accumulates [53]. Because of the multiple copies of mitochondrial genomes per cell, detecting and characterising a single mutation is rather difficult [54]. A particular threshold, where the mutation level is so high as to interrupt normal functioning, was not defined. Mutant mtDNA levels should be around 60–90% [55] to exceed repair mechanism capacity [56]. Most probably, this threshold is specific for every mutation type and cell-type, tissue, organs, or even person [57,58,59,60]. Many experiments have proved a direct connection between mtDNA mutations load, lifespan and rate of ageing [61,62,63]. The same mechanism was also identified in humans, where it was shown to be involved in developing Parkinson’s disease [64,65].

### 2.2. Mitophagy Dysregulation and Defects

In addition to the ROS-mediated damage of the mtDNA and proteins, the age-dependent decline in the removal of malfunction mitochondria influences the normal functioning of mitochondria. Selective removal of damaged proteins and mitochondria parts occurs through mitophagy [66]. Damaged proteins are ubiquitinated and accumulated on the outer mitochondrial membrane. Ubiquitination is catalysed by the E3-ubiquitin ligase Parkin, activated by PTEN-inducible putative kinase 1 (PINK1) [67]. Further, PINK1/Parkin-mediated mitophagy acts as a positive feedback amplification cascade [68].

Malfunction mitophagy and mitochondria dynamics have been connected to many human diseases, most importantly neurodegenerative diseases like Parkinson’s disease (PD), Alzheimer’s disease (AD), and Huntington’s disease (HD) [69,70]. Not surprisingly, the first connection between mitophagy and PD was established when mutations in the mitophagy-regulating genes were found. The primary hereditary form of PD has mutations in the *PARK6* and *PARK2* genes, encoding PINK1 and Parkin, respectively [71]. Interestingly, deletion of the autophagy genes leads to the accumulation of abnormal mitochondria and a rise in ROS production [72]. Additionally, a positive correlation between mitophagy rate, mitochondrial health and longevity have been established on many model systems. Thus, stimulation of mitophagy (chemically or with genetic manipulation) leads to increased longevity in *C. elegans* [73], Drosophila [74,75], and humans [76].

Similarly, chemical treatment was studied on zebrafish retina (resveratrol [77]), rat hippocampus explant cultures, SKNSH human neuroblastoma cells (Kisspeptin-10 [78]), mice heart (Kanglexin [79]), and C. elegans (tomatidine [80]). Urolithin A has shown promising results in C. elegans, rodents [81] and human [82] studies.

Thus, these studies imply that mitophagy is a target for many mutations, leading to the development of a life-threatening disease; and specific treatment could influence mitophagy to provide positive effects on age-related diseases and improve the quality of life of older people.

### 2.3. Criticism: The Connection between Lifespan and ROS; Beneficial Effects of Mild Stress

The main criticism of the ROS-mediated theory of mitochondria damage and subsequent ageing is based on the experiments breaking the connection between ROS level and lifespan. In particular, overexpression of the CuZn superoxide dismutase does not prolong the lifespan of many species by reducing ROS [83,84], but through a more complex pathway that includes ER-stress response factors [85]. Similarly, the reduction of antioxidant enzymes increased MtDNA damage but did not influence lifespan [86,87,88]. Also, a high level of oxidative stress was noticed in some long-living species [89,90]. Altogether, this suggests that a mild level of oxidative stress could be beneficial for organisms, while, in the broader scope, the ROS-mediated theory of ageing requires further research to update [91].

## 3. Inflammaging

Inflammaging theory is not new; many aspects of the relations between inflammation, development of age-related diseases and ageing have been suggested by many researchers before, while in a modern form, this theory was postulated in the 1990s [reviewed in [92]. This specific role was also given to the status of the immune system, which, due to the influence of age-related processes (immunosenescence), was unable to timely deactivate the inflammation and transmit it to the chronic form [93]. However, the proper terminology is still a matter of discussion while scientists mainly accept the main principle of inflammaging: it is an age-dependent increase in one’s pro-inflammatory status that influences lifespan and has associated pathological processes (diseases) [16]. Furthermore, we will characterise the most crucial aspects in more detail.

Pathogen attack, infection, or tissue injury could trigger normal inflammation. A fine-tuned signalling network regulates the shift from a pro-inflammatory state to a highly active inflammation state. Usually, inflammatory responses vanish as only threat or injury is removed (so-called resolved inflammation). In the case of non-resolved inflammation, some unknown factors are keeping on, providing low-level and long-lasting stimulation of the inflammation process in the cell or tissue [94].

### 3.1. Shift from Inflammation to Chronic Inflammation

Pro-inflammatory cytokines are the main factors responsible for a shift from normal inflammation to chronic inflammation and subsequent pathological inflammaging [95]. Experimental evidence from animals suggests an involvement of IL-1β, IL-4, IL-10, IL-17, IFN-γ, NF-κB, TNF-α into the chronic inflammation development [96,97,98]. It was shown in elderly people that high levels of IL-6 and TNF-α are associated with disabilities and mental and physiological disorders [99,100]. As a result of several large-scale studies, the serum level of IL-6 was suggested as a reliable general marker of inflammaging and some specific diseases and infections like COVID-19 and cancer [101,102]. The promoter regions of cytokines are polymorphic in the human population that could cause a regional difference in reaction and susceptibility to the development of age-related diseases, inflammaging and lifespan [103,104,105]. 174 G > C polymorphism in the *IL-6* gene is associated with a high risk of heart failure in patients with obesity and heart diseases [106]. A study of the Chinese Han population suggests higher susceptibility to the development of knee osteoarthritis for individuals with IL-6 rs12700386 polymorphism. Adverse environmental factors (smoking and drinking) in combination with the rs12700386 genotype have shown even higher osteoarthritis risk, establishing a solid connection between the gene and environment [107]. As was demonstrated in another study, IL-6-174 G/C polymorphism was not connected to the ophthalmic diseases, while the GC genotype of IL-6-174 G/C was associated with proliferative diabetic retinopathy. Also, a higher intraocular level of IL-6 was defined among ocular disease patients [108]. The polymorphism of four genes examined in the North-Italian population (*IL-6* (G > C, rs1800796), *IL-10*-1082 (G > A, rs1800896), *TNF-α*-308 (G > A, rs1800629), and *TGFβ1* codon 10 (T > C, rs1800471)) was connected with an increased duration of inflammation and cancer risk [109].

Several pro-inflammatory cytokines, like TNF-α and interferons, were shown to cause cellular senescence and enhance inflammaging. Such senescence was studied in many cell-types [110,111], including stem cells [112,113], and driven by different ROS-based pathways (such as NF-κB [114], JAK/STAT [115], TGFβ/Smad [116]), launching a positive feedback loop that supports the further release of ROS and some cytokines [115].

From a general point of view, an imbalance of pro-inflammatory/anti-inflammatory cytokines could explain the inflammaging-mediated ageing. On the other side, several large-cohort studies suggested that longevity could be associated with the inflammaging process [117,118,119]. However, while those results have been obtained on relatively closed centenarians’ groups (Japanese, Italians and Greeks, respectively), the exact molecular mechanism is not proven and requires further investigation.

### 3.2. Role of mtDNA Mutations in Inflammation

In addition to the role of mitochondrial mutations and mitophagy in many life-threatening diseases, discussed in Section 2.1, the same violations could also influence other cell types (including immune cells [120] and alter their functionality, with further effect on inflammation and inflammaging processes.

Mutation m.3243A > G in *mtTL1* gene, encoding tRNA^Leu-UUR^, was identified in 80% of patients with MELAS maternally inherited mitochondrial disorder. MELAS (mitochondrial encephalomyopathy, lactic acidosis, and stroke-like episodes) is a rare genetic disorder characterised by the brain, nervous system and muscles symptoms (temporary muscle weakness, hallucinations, seizures, abdominal pain, difficulty understanding, thinking or speaking and many others) with no cure available. Malfunction of the tRNA^Leu-UUR^ leads to the disassembly of the electron transport chain, subsequent defective oxidative phosphorylation, and energy production. At the final stages, those events reduce the production of ATP and accumulate lactate, causing lactic acidosis. Such events are incredibly stressful for energy-demanding cells, like neurons and myocytes [121]. Recently it was shown on endothelial cells delivered from MELAS syndrome patients that they have a high level of ROS and ox-LDL (oxidised-low-density lipoprotein). Also, the basal level of isoform b of VCAM-1 was high [122]. *VCAM-1* is a crucial inflammation gene responsible for leucocyte-endothelial cell adhesion before leucocytes transmigrate through vascular walls [123].

M.3243A > G mutation showed a high level of autophagy and a deficit of mitophagy on MELAS-patient delivered induced pluripotent stem cells (iPS). Under oxidative stress challenge, bulk macroautophagy was increased, with further accumulation of toxic autophagosomes and autolysosomes and subsequent lower cell viability [124].

Rett syndrome, a crucial neurodevelopmental disorder, is associated with mutations in the *MECP2* (methyl-CpG-binding domain-containing-protein 2) gene, clearly linked with abnormal mitochondrial dynamics, morphology and mitophagy [125]. Mitochondria malfunction leads to imbalanced redox status, high cytokine production and aberrant immune response, resulting in chronic inflammation [126].

Recent experiments on a mice model have identified age-related V338Y amino acid exchange in the MRPS5 (a mitochondrial ribosomal protein) as the ram (ribosomal ambiguity) mutation, leading to ribosomal proteins mistranslation. In the skeletal muscle, MrpS5 V338Y mutation resulted in impaired oxidative phosphorylation, higher ROS and bioactive lipids production, and enhanced inflammation [127].

Recently, mitochondrial tRNA^Thr^ 15927G > A mutation was linked to the pathogenesis of coronary artery disease. M.15927G > A mutation causes lower respiration efficiency, diminished membrane potential and higher ROS production on a cybrid cell culture. On the organism level, such mutation might be responsible for the inflammatory vascular reactions leading to cardiovascular diseases [128]. A novel mitochondrial tRNA^Gln^ m.4349C > T mutation was identified in the blood, urinary sediments and muscle samples of a patient with encephalopathy, epilepsy, and deafness. Similarly to other mitochondrial tRNA mutations, tRNA^Gln^ m.4349C > T has lower efficiency of the respiratory chain complex, high ROS level and decreased mitochondrial membrane potential, leading to premature cell senescence [129].

T6459C mutation of the *mtCO1* gene, identified in the Chinese Han population, was linked to the higher genetic susceptibility to sepsis. *mtCO1* encodes a functional subunit of COX protein that can fix ROS. Under normal conditions, biochemical parameters were close to the non-mutant group. However, after 6 h of lipopolysaccharide stimulation (simulation of the pathogen infection environment), the mutation-currying group has shown higher ROS production, lower levels of ATP and mitochondrial membrane potential, higher apoptosis rate. In total, those results suggest the prolonged release of the pro-inflammation mediators due to mitochondria injury and increased production of ROS [130].

Thus, the mitochondrial mutation, high ROS level, unbalanced nutrient supply and pathogens could affect mitophagy, directly and indirectly, engaging many physiological pathways and influencing the inflammation and shifting it to chronic status.

### 3.3. The Theory of Oxidation-Inflammation

A close correlation between mitochondria functionality, generated oxidative stress, the status of the immune system, severity of inflammation and ageing has been known to exist for a long time [131]. The modern theory, combining those processes, isoxidation-stress-mediated inflammaging [132]. Mitochondria play the primary role in this theory, where the original oxidative stress appears and leads to a further disturbance on the cellular level. A positive point of this theory, supported by many researchers, suggests that an adequate supply of cells with antioxidants may normalise the redox state, improve the immune system’s efficiency, extend lifespan, and lower the disease burden. Considerable research data has suggested that calorie restriction is the simplest type of intervention, with proven positive effects. Many aspects of calorie restriction (CR) were studied, and we can redirect readers to a specific topic of their interest: nutrition habits and inflammation status [133], the role of CR in tumour progression and inflammaging [134], autophagy and inflammation [135], microbiota and inflammation [136], cognition [137] and inflammaging [138]. A newly emerging area in the inflammaging research implies the application of artificial and natural compounds that mimic calorie restriction [139,140].

**Figure 1:**
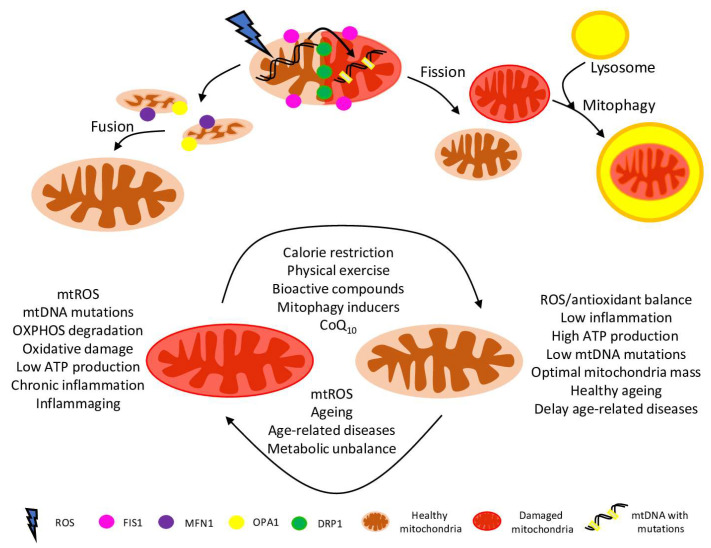
Schematic representation of the role of mitochondria in healthy ageing. Mitochondrial dynamics are the coordinated fusion/fission events driven by complex machinery, involving Mfn, OPA1, Drp1, Fis1 and many other proteins. Mitochondrial fusion (interconnecting organelles) promotes mtDNA mixing and enhances bioenergetic efficiency. Mitochondrial fission is organelle segregation: equal distribution between daughter cells, healthy and damaged parts separation. Aged, damaged, and defective mitochondria are targeted for degradation via mitophagy. Mitochondrial health is crucial for delaying ageing and age-related diseases, including many metabolic disorders. Also, dysfunctional mitochondrial release many damage signals to the cytosol, which are leading to the release of inflammatory cytokines and causing chronic inflammation associated with ageing and age-related diseases. However, some activities, compounds and procedures (such as CoQ_10_ supply, calorie restriction and physical exercise) could reverse the damage and ameliorate mitochondria via mitophagy and de novo biogenesis.

Chronic oxidative stress impacts every cell type, whereas the most pronounced effect can be observed in active, energy-demanding cells responsible for regulation and homeostasis, such as immune, nervous and endocrine cells [141,142,143]. Not surprisingly, many chronic inflammation-based diseases manifest in elderly people, significantly decreasing lifespan, quality of life, and increasing susceptibility to many accompanying diseases (Summarised in Figure 1). In the next section, we will update the role of inflammaging in the most common neurodegenerative diseases such as Alzheimer’s and Parkinson’s.

### 3.4. Noncanonical mtDNA-Mediated Inflammation

MtDNA itself is a circular molecule of double-stranded DNA that is recognised as “foreign” by the immune system and that triggers various inflammatory pathways. The hypomethylated status of mtDNA could explain such an effect, where unmethylated CpG motifs would be similar to bacterial DNA and could potentially activate pattern recognition receptors such as TLR9 (Toll-Like Receptor 9) [144]. Additionally, the key cytosolic DNA sensor cGAS (Cyclic GMP-AMP Synthase) could recognise RNA:DNA hybrids and long stretches of single-stranded DNA, typical for mitochondrial DNA replication and transcription processes [145].

The release of mtDNA from mitochondria and subsequent activation of TLR9, cGAS and inflammasomes could occur during many cellular processes, including infection, neurodegeneration and cell death. The pioneering research in 2004 [146] demonstrated that local inflammation and arthritis could be experimentally induced by the injection of mtDNA into mice joints. Also, induced inflammation depended on the presence of oxidatively damaged bases in the mtDNA.

Nowadays, mtDNA is associated with the pathogenesis of several inflammatory diseases, such as diabetes, sickle cell disease, non-alcoholic steatohepatitis, lupus-like disease, Aicardi–Goutières syndrome, myocardial infarction and Parkinson’s disease. Therefore, we wish to redirect interested readers to the recent reviews [147,148].

## 4. Alzheimer’s Disease

AD is a common chronic neurodegenerative disease affecting approximately 50 million people in 2020 [149], with the most prominent symptoms similar to dementia and behavioural and memory problems. Despite more than 100 years of intensive studies (Alois Alzheimer described the first case in 1907), the understanding of the cause of AD is still not certain. Frontal and limbic cortices microglia inflammation is the common source of pro-inflammatory and oxidative stress, and is mostly associated with neurofibrillary tangles (NFT) and amyloid beta peptide (Aβ) deposits [149]. At the latter stages, AD is associated with higher and wider Aβ deposition, dysregulation of T-cell responses, increased lipid peroxidation and lower levels of plasma antioxidants [150]. Also, AD patients without dementia histories have a high level of Aβ/NFT but much lower levels of pro-inflammatory markers [151]. On the other side, the level of anti-inflammation cytokines is also crucial for the disease progress and could be connected to a particular genotype and degree of the immune system’s efficiency [152,153]. We could also redirect readers interested in the specific role of mitophagy in AD development to a recent review [154].

Several studies suggest a connection between pro-inflammatory cytokines and the Aβ peptide level. For example, injections of IFNγ up-regulate Atg5 and Atg7 expression, thus enhancing autophagy and, subsequently, lowering Aβ toxicity and decreasing the Aβ plaque load in the cortex and hippocampus [155]. Similarly, IL-10 and IL-12 have been defined as protective cytokines in another study [156], where systemic inflammation was found to be associated with hippocampal atrophy and cerebrospinal fluid protein levels in AD patients. One study defines IL-9 as a reliable marker for AD-related changes in African Americans but not Caucasians [157], while the exact genetic or environmental reasons and consequences for those observations are now known and require further investigation. Systemic levels of the cytokines IL-6 and IL-10 were higher in AD patients, while other cytokines did not correlate with neuroinflammation [158]. IL-10 is one of the major cytokines, playing a crucial role in many neurodegenerative diseases [159]. IL-33 is one of the minor cytokines, most likely playing an anti-inflammatory role and preserving cognitive functions in non-AD patients, while AD patients have a lower level of IL-33 and cognitive decline [160].

Effective mitophagy in microglia cells plays a crucial role in the removal of neurotoxic components, regulation of the pro-inflammation/anti-inflammatory cytokines balance, and general immune reactions. As was recently shown on the APP/PS1 mouse AD model [161], PINK1- and parkin-dependent mitophagy in microglia is responsible for the elimination of pro-inflammatory cytokines and intracellular Aβ. The revealed mechanism suggests adverse AD effects not only on neural cells but also on peripheral tissues. Mitophagy stimulation reverses cognitive symptoms and decreases extracellular Aβ plaques and the degree of neuroinflammation in several Aβ and tau AD animal model systems, suggesting mitophagy as a potential therapeutic target [161].

Similarly, aged mutant APP mouse (an AD model, expressing transgenic human amyloid-beta precursor protein (APP)), has shown altered levels of mitochondrial fission/fusion proteins, decreased levels of autophagy and mitophagy marker proteins [162]. Closer examination of APP mice hippocampal tissues revealed higher mitochondrial numbers but lower mitochondrial length, accompanied by defective mitochondria biogenesis, structure and dynamics, and cognitive degradation.

Recent results obtained in sporadic and familial AD patients suggest a tighter connection between ER and mitochondria contact sites (MERCS) in brain tissue and primary neurons [163]. Also, in AD animal models, increased levels of Aβ was associated with altered mitochondria functions and autophagosome formation. Altogether, this suggests a new role of MERCS as a self-cleaning mechanism aimed at removing toxic Aβ aggregates by quicker autophagosome formation and increased mitochondrial energy production [163].

As shown on the mouse AD model, accumulation of mutant APP and Aβ in hippocampal neurons leads to higher mitochondrial numbers, reduced mitochondrial length and a general decline of cell survival. Also, mitochondrial biogenesis, dynamics, mitophagy rate and general functions have been reduced [164].

## 5. Parkinson’s Disease

Parkinson’s disease (PD) is the second (after AD) most prevalent neurodegenerative disease; approximately 1% of the worlds’ population over the age of 60 have been diagnosed with it [165]. Slowly emerging symptoms include mainly motor functions (tremor, slowness, walking, rigidity), but cognitive and behavioural indications are also possible [166]. Despite almost two centuries of intensive study, the exact cause for PD is unknown, although it is likely that both inherited and environmental factors are involved [167]. Several recent excellent reviews described the close connection between mitochondria functions, effective mitophagy and autophagy in PD development, its pathogenesis, and therapy [168,169,170]. Furthermore, we will highlight the most important research in line with the inflammaging theory, mtDNA mutations and mitophagy.

Recent data further supported the role of age-dependent neuroinflammation. Two important features were associated with biallelic parkin/PINK1 mutations in German and Italian cohort patients. Firstly, such patients have higher levels of IL-6, which suggests using IL-6 as a reliable disease progression marker. Secondly, this mutation was linked with a high level of circulating cell-free mtDNA in serum, causing further progress and spreading neuroinflammation. Those data suggest that the application of NSAIDs (general unspecific therapy) and targeted anti-IL-6 antibodies (specialised treatment) may have a positive effect on Parkinson’s disease progress [171].

Insufficient PINK1-mediated mitophagy in the brain causes a lower level of dopamine and the release of cytokines (like TNF-α, IL-1β) by astrocytes and microglia [172]. A sufficient level of PINK1 may restrict neuroinflammation, ROS production and cell death, suggesting PINK1 protection from proteolysis as a promising strategy in PD treatment. Recently, a small molecule, BC1464, has been shown to limit PINK1 degradation by the ubiquitin-proteasome system, thus preventing mitochondrial damage. Neuroprotective properties of BC1464 have been confirmed on human primary cortical neurons, neuroblastoma cells and several PD patient-derived cell cultures, suggesting its application in PD treatment [173]. The application of the anticancer drug gemcitabine, which acts presumably via the MUL1 (mitochondrial ubiquitin ligase) pathway, leads to PINK1 stabilisation [174].

Degraded dopaminergic neurons in the *substantia nigra pars compacta* are the main diagnostic trait of PD. The recent paper also describes the implication of PD development in the cerebellum, which is responsible for some motor-related functions [175]. We have found increased levels of pro- (I-309, TNF and IL-1β) and anti-inflammatory (IL-1ra) cytokines in cerebellar mitochondria. This paper suggests that the pathophysiological manifestation of PD is much broader and not limited only to the *substantia nigra brain regions.*

In total, we could conclude that mitochondria health plays a crucial role in AD and PD development, closely connecting inflammation status, redox/antioxidants balance, cell energy demand and toxin removal.

## 6. Conclusions

Inflammaging is a modern concept of the ageing process based on long-lasting system inflammation. Inflammaging defines the pace of ageing, lifespan and quality of life of an aged population and is highly related to major human diseases, such as AD, PD, heart diseases, multiple sclerosis, atherosclerosis, cancer, type II diabetes, and many others [176]. Chronic, subclinical inflammation may be used as a marker for screening of risk groups and particular cytokinesas a therapeutic target when the specific disease is defined.

Despite intensive research, the exact molecular mechanism of inflammaging is still not known. Here we have discussed some therapeutic interventions that could be promising in combatting inflammaging. For example, mitophagy, as the basic cellular process, could be sped-up and improved by genetic manipulations and medicines. Cytokine-specific antibodies applied in particular tissues or organs could appose chronic inflammation. In a very optimistic scenario, such intervention could serve as a universal tool, amending many diseases and prolonging lifespan. For now, unfortunately, such a miracle tool is still missing; however, as we discussed, some effective methods have been found, such ascalorie restriction and regular physical exercise.

Thus, we could conclude that healthy mitochondria are crucial for proper cellular functioning. Further research on the fundamental molecular level and clinically controlled pharmacologic modulation therapy are required.

## Data Availability

Not applicable.

## Abbreviations

Aβ	Amyloid Beta Peptide
AD	Alzheimer’s disease
APE1	apurinic/apyrimidinic endonuclease
APP	amyloid-beta precursor protein
BER	base excision repair
COX	cytochrome C oxidase
CR	calorie restriction
HD	Huntington’s disease
HSP	heat shock proteins
IFN-γ	Interferon-gamma
IL-4	Interleukin 4
iPS	induced pluripotent stem cells
JAK	Janus Kinase
MECP2	methyl-CpG-binding domain-containing-protein 2
MELAS	Mitochondrial encephalomyopathy, lactic acidosis and stroke-like episodes
MERCS	mitochondria and ER contact sites
MRPS5	mitochondrial ribosomal protein
MQC	mitochondrial quality control
NF-κB	nuclear factor kappa-light-chain-enhancer of activated B cells
NFT	neurofibrillary tangles
NSAIDs	non-steroid-anti-inflammatory-drugs
OXPHOS	oxidative phosphorylation system
PD	Parkinson’s disease
PINK1	PTEN-inducible putative kinase 1
ram	ribosomal ambiguity
ROS	reactive oxygen species
STAT	signal transducers and activators of transcription
TGF-β	Transforming growth factor-beta
TNF-α	tumour necrosis factor-alpha
UPRmt	mitochondrial unfolded protein response
UPS	ubiquitin/26S proteasome system
VCAM-1	vascular cell adhesion molecule-1
